# Supplementary material for: A novel 14-3-3θ phosphomimetic mouse model demonstrates social dominance defects
Source: Biol Open. 2025 Jun 10;14(6):bio061963. doi: 10.1242/bio.061963 (PMC12182864; doi:10.1242/bio.061963)
Supplement: Supplementary information [file biolopen-14-061963-s1.pdf]

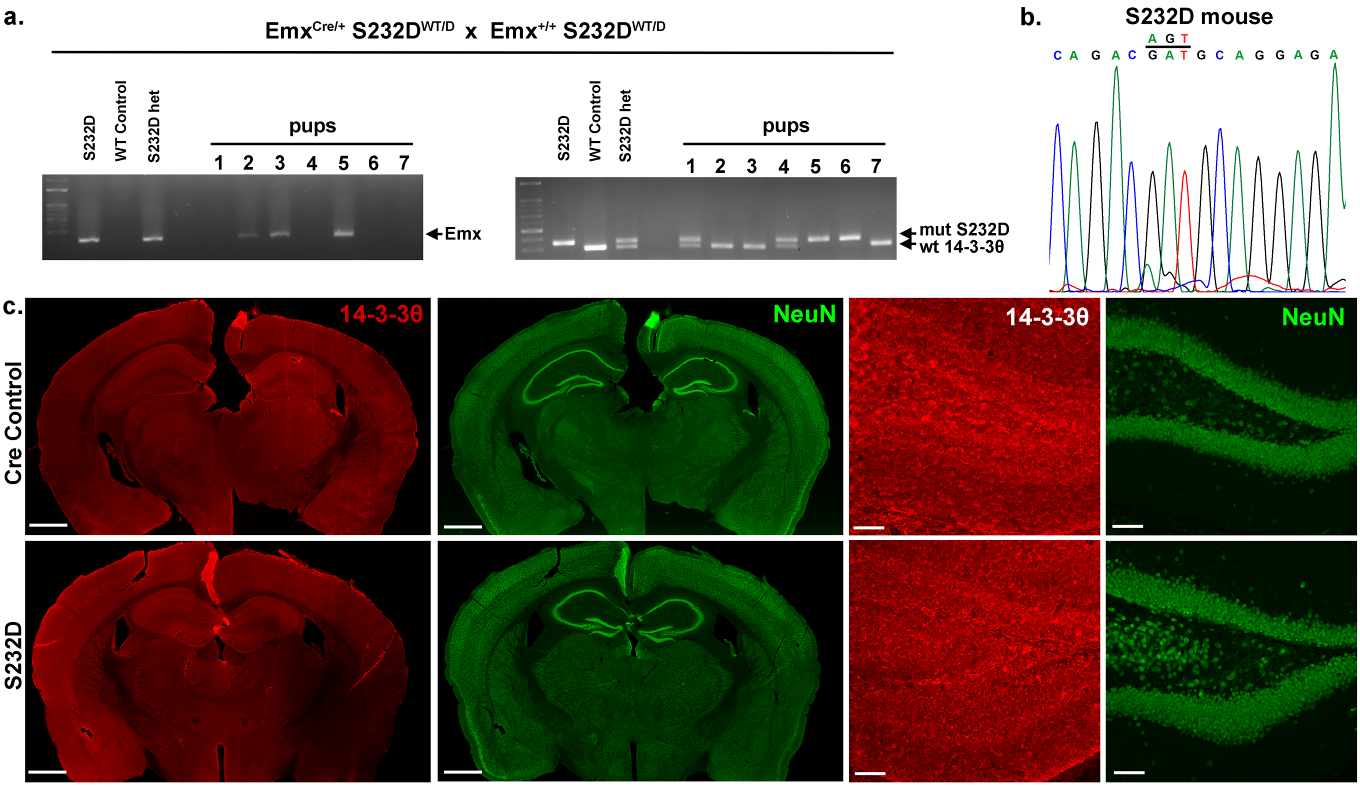


## Fig. S1. S232D Conditional Knock-in Mouse Lines

**A**. Genotyping of mice resulting from crossing Emx1-Cre -/- S232D +/- and Emx1-Cre +/- S232D +/- mice.

**B**. Sanger sequencing of cDNA generated from RNA extracted from an S232D mouse cortex, confirming a change from serine (AGT) to aspartic acid (GAT) at amino acid 232.

**C**. Immunohistochemistry for 14-3-3θ and NeuN in brains of 14month old Cre control (top) and S232D (bottom) mice. Scale bars for whole brain images = 1 mm. Scale bars for zoomed in hippocampal images = 100 um.

Biology Open • Supplementary information

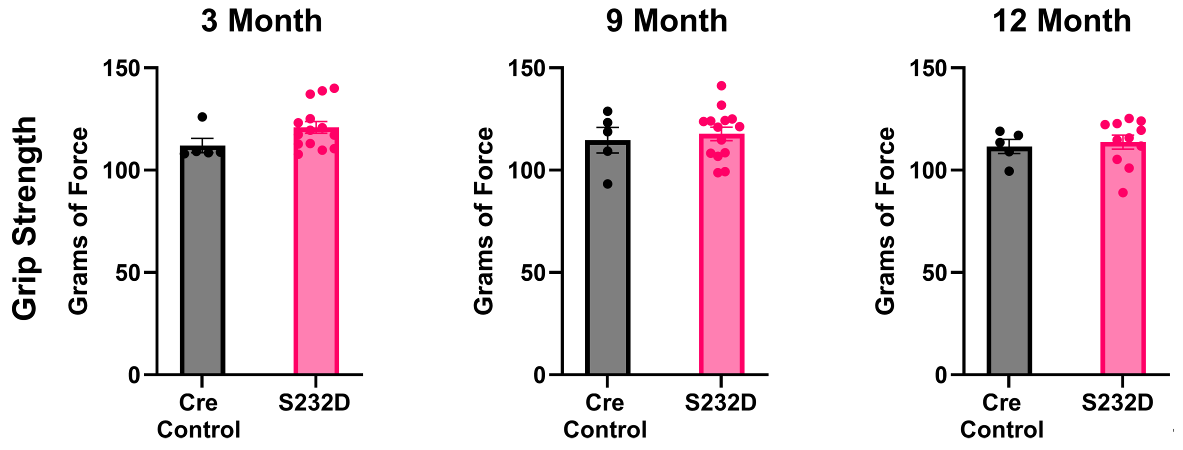


**Fig. S2. S232D and Cre control mice perform similarly in grip strength test**

Quantification of grip strength at three, nine, and 12 months from the first cohort of mice. n=5-14 mice per group. Error bars represent SEM

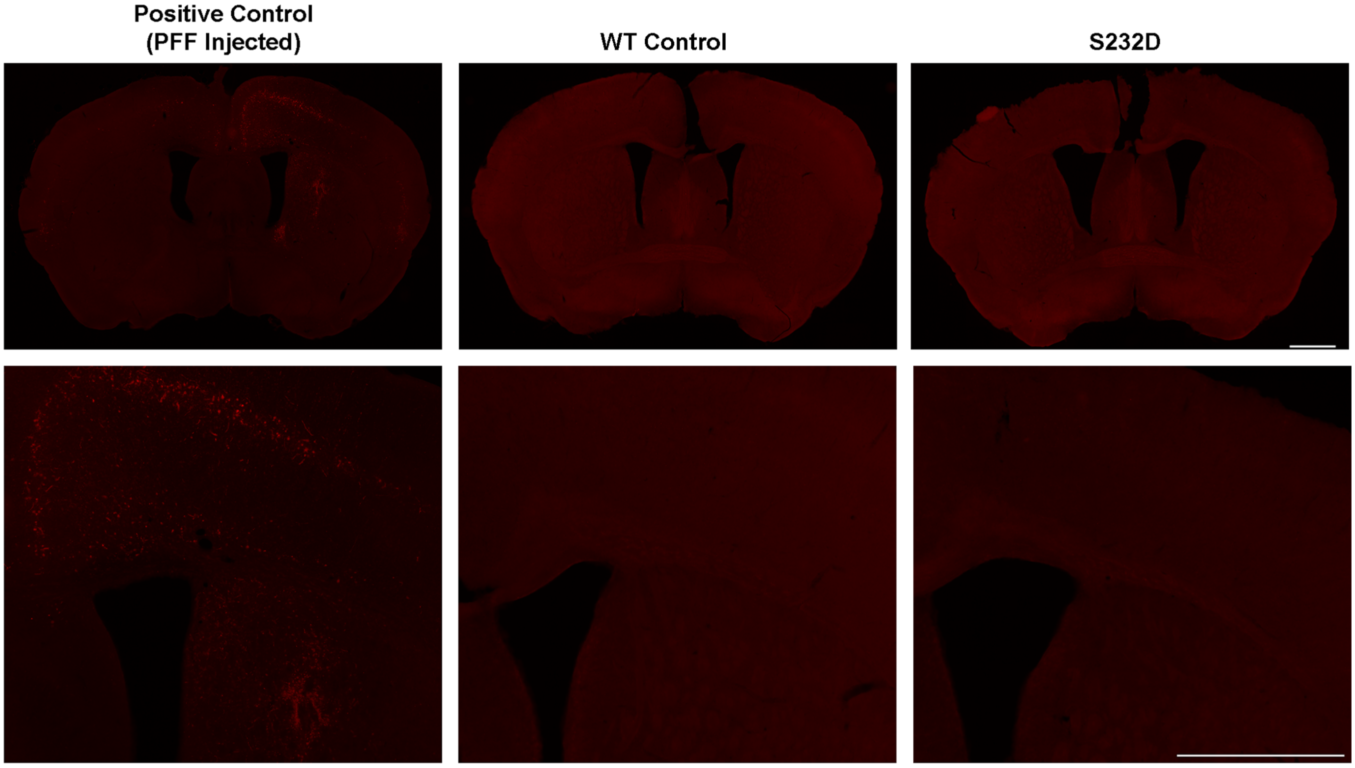


**Fig. S3. S232D mice do not exhibit characteristic phospho-asyn inclusions.**

Representative whole brain and zoomed images of psyn staining of a preformed fibril (PFF)-injected positive control mouse, WT control mouse, and S232D mouse. The positive control mouse exhibits positive staining for psyn, while the WT control and S232D mice do not.
